# Volumetric Integrated Classification Index: An Integrated Voxel-Based Morphometry and Machine Learning Interpretable Biomarker for Post-Traumatic Stress Disorder

**DOI:** 10.1007/s10278-024-01313-5

**Published:** 2024-11-04

**Authors:** Yulong Jia, Beining Yang, Haotian Xin, Qunya Qi, Yu Wang, Liyuan Lin, Yingying Xie, Chaoyang Huang, Jie Lu, Wen Qin, Nan Chen

**Affiliations:** 1 Department of Radiology and Nuclear Medicine, Xuanwu Hospital, Capital Medical University, Beijing, 100053 China; 2 Beijing Key Laboratory of Magnetic Resonance Imaging and Brain Informatics, No. 45 Chang-chun St, Beijing, 100053 Xicheng District China; 3 Department of Radiology and Tianjin Key Lab of Functional Imaging, Tianjin Medical University General Hospital, 154 Anshan Road, Tianjin, 300052 Heping District China

**Keywords:** PTSD, Biomarker, Voxel-based morphometry, Machine learning, Volumetric integrated classification index

## Abstract

PTSD is a complex mental health condition triggered by individuals’ traumatic experiences, with long-term and broad impacts on sufferers’ psychological health and quality of life. Despite decades of research providing partial understanding of the pathobiological aspects of PTSD, precise neurobiological markers and imaging indicators remain challenging to pinpoint. This study employed VBM analysis and machine learning algorithms to investigate structural brain changes in PTSD patients. Data were sourced ADNI-DoD database for PTSD cases and from the ADNI database for healthy controls. Various machine learning models, including SVM, RF, and LR, were utilized for classification. Additionally, the VICI was proposed to enhance model interpretability, incorporating SHAP analysis. The association between PTSD risk genes and VICI values was also explored through gene expression data analysis. Among the tested machine learning algorithms, RF emerged as the top performer, achieving high accuracy in classifying PTSD patients. Structural brain abnormalities in PTSD patients were predominantly observed in prefrontal areas compared to healthy controls. The proposed VICI demonstrated classification efficacy comparable to the optimized RF model, indicating its potential as a simplified diagnostic tool. Analysis of gene expression data revealed significant associations between PTSD risk genes and VICI values, implicating synaptic integrity and neural development regulation. This study reveals neuroimaging and genetic characteristics of PTSD, highlighting the potential of VBM analysis and machine learning models in diagnosis and prognosis. The VICI offers a promising approach to enhance model interpretability and guide clinical decision-making. These findings contribute to a better understanding of the pathophysiological mechanisms of PTSD and provide new avenues for future diagnosis and treatment.

## Introduction

Post-traumatic stress disorder (PTSD) is a complex mental health condition rooted in individuals’ traumatic experiences, often having a long-term and profound impact on sufferers’ psychological health and quality of life [1]. PTSD manifests through a variety of symptoms including intrusive memories, avoidance behaviors, negative changes in thought and mood, and heightened arousal reactions [2]. This symptom diversity makes diagnosis particularly challenging. Traditional diagnostic methods, such as clinical interviews and standardized questionnaires (e.g., the Clinician-Administered PTSD Scale, CAPS) [3], rely heavily on subjective reports and the clinical judgment of healthcare providers. This subjectivity can lead to variability in diagnosis and may not fully capture the complexity of PTSD symptoms. Moreover, some patients may be reluctant to disclose their symptoms due to stigma or distrust of medical professionals, further complicating accurate diagnosis [4]. This reliance on subjective reports introduces variability into diagnostic outcomes and may fail to fully capture the complexity of the disorder. Neuroimaging has emerged as a powerful tool in overcoming these challenges, providing objective, measurable insights into the brain’s structure and function [5]. Unlike traditional methods that depend heavily on patient self-reporting and clinical interpretation, neuroimaging allows clinicians and researchers to visualize and quantify the biological underpinnings of mental disorders [6]. This offers a more consistent and precise diagnostic approach, improving the ability to detect abnormalities that may be invisible through psychological assessments alone. Given the potential of neuroimaging, researchers [7–9] have increasingly turned to these techniques to identify biomarkers that can enhance diagnostic precision and guide treatment strategies. Large, publicly available neuroimaging databases, such as the Alzheimer’s Disease Neuroimaging Initiative (ADNI) and its extension, the Department of Defense Alzheimer’s Disease Neuroimaging Initiative (ADNI-DoD), play a crucial role in this effort. These databases provide high-quality, standardized imaging data across different populations and conditions, facilitating the development and validation of neurobiological markers. Utilizing such resources enables comprehensive analyses with large sample sizes, ensuring more reliable and generalizable findings. Additionally, standardized imaging protocols across these databases allow for rigorous comparisons, helping to establish biomarkers that can be applied to clinical settings for more accurate PTSD diagnosis and personalized treatment.

Over the past decade, with the development of neuroimaging, Voxel-Based Morphometry (VBM) has made significant strides in studying brain structure changes related to PTSD. Through a comprehensive meta-analysis of a series of MRI studies, researchers found that brain structures of individuals with PTSD exhibit distinct differences compared to healthy controls [10–12]. For instance, PTSD patients tend to manifest reduced hippocampal volumes, potentially affecting their memory capabilities, particularly memories associated with traumatic events. The anterior cingulate cortex, a brain area associated with emotional regulation, also exhibits volumetric changes. Additionally, volumetric changes are noted in the prefrontal cortex, a brain area involved in decision-making and social behavior, and the amygdala, a brain area related to fear response and emotional processing, among individuals with PTSD [12]. The application of these methods has aided researchers in understanding how the brain responds as an integrated network to PTSD, revealing how brain regions interact with each other to collectively contribute to PTSD. However, the heterogeneity of PTSD presentations among individuals, including differences in trauma types, severity, and comorbidities, complicates the interpretation of research findings. Additionally, the field is continually evolving, with advancements in technology and methodologies, which can impact the consistency of reported results. Therefore, addressing these issues is crucial to further our understanding of the neural mechanisms underlying PTSD.

The application of machine learning in the field of neuroimaging has grown rapidly, particularly in the development of imaging features for individual brain function and structure. Machine learning methods in clinical neuroimaging have revealed imaging features of various diseases and disorders, such as Alzheimer’s disease, brain development and aging, schizophrenia, and autism, among others. In cognitive neuroscience, machine learning methods hold promise for identifying functional fingerprints of individual brains and brain states [13]. Progress has also been made in PTSD research. Researchers are using machine learning methods to differentiate between the brain structures of PTSD patients and healthy individuals. By analyzing the local features, connectivity, and network properties of brain regions in the fear circuit, potential methods for revealing early signs of PTSD have been identified [14].

There is also a study that has demonstrated that machine learning models can effectively differentiate PTSD patients based on structural changes in key brain regions such as the prefrontal, temporal, and parietal regions, the cerebellum, and the putamen, which are areas implicated in the pathophysiology of PTSD [15].

However, while these machine learning models provide diagnostic and prognostic value, they also bring the issue of interpretability. The “black box” nature of the models makes their outputs challenging to interpret, potentially limiting their application in clinical practice [16, 17]. To address the “black box” issue, several methods have recently been proposed to aid users in interpreting machine learning predictions. For instance, a recent study introduced a neuroimaging biomarker called the “functional striatal abnormalities (FSA) score,” which is built upon the classification distance of the separating hyperplane by a support vector machine classifier. This approach demonstrated significant improvements in the biological significance and classification accuracy of schizophrenia, achieving an accuracy rate of over 80% in distinguishing patients from healthy controls [18]. Additionally, the SHapley Additive exPlained (SHAP) framework has been recently proposed to assign each feature an additive importance value for specific predictions within a single model [19]. This method enhances the interpretability of machine learning models by providing clear insights into which features most influence the model’s predictions, thereby improving transparency and trust in clinical applications. These advancements have proven highly effective in other psychiatric disorders [20], significantly enhancing model interpretability. However, these methodologies have not yet been widely applied to PTSD classification models, presenting a promising opportunity for future research to leverage these techniques for more precise and explainable PTSD diagnostics. In summary, this study is grounded on the formidable foundations of VBM neuroimaging techniques, further integrating machine learning methods and model interpretability techniques, aiming to discover and validate brain structural changes associated with PTSD. The use of machine learning interpretability tools like SHAP will enhance our understanding of the predictive nature of models and provide transparent and accurate diagnostic criteria for clinical application.

## Materials and Methods

### Participant Selection

**Fig. 1:**
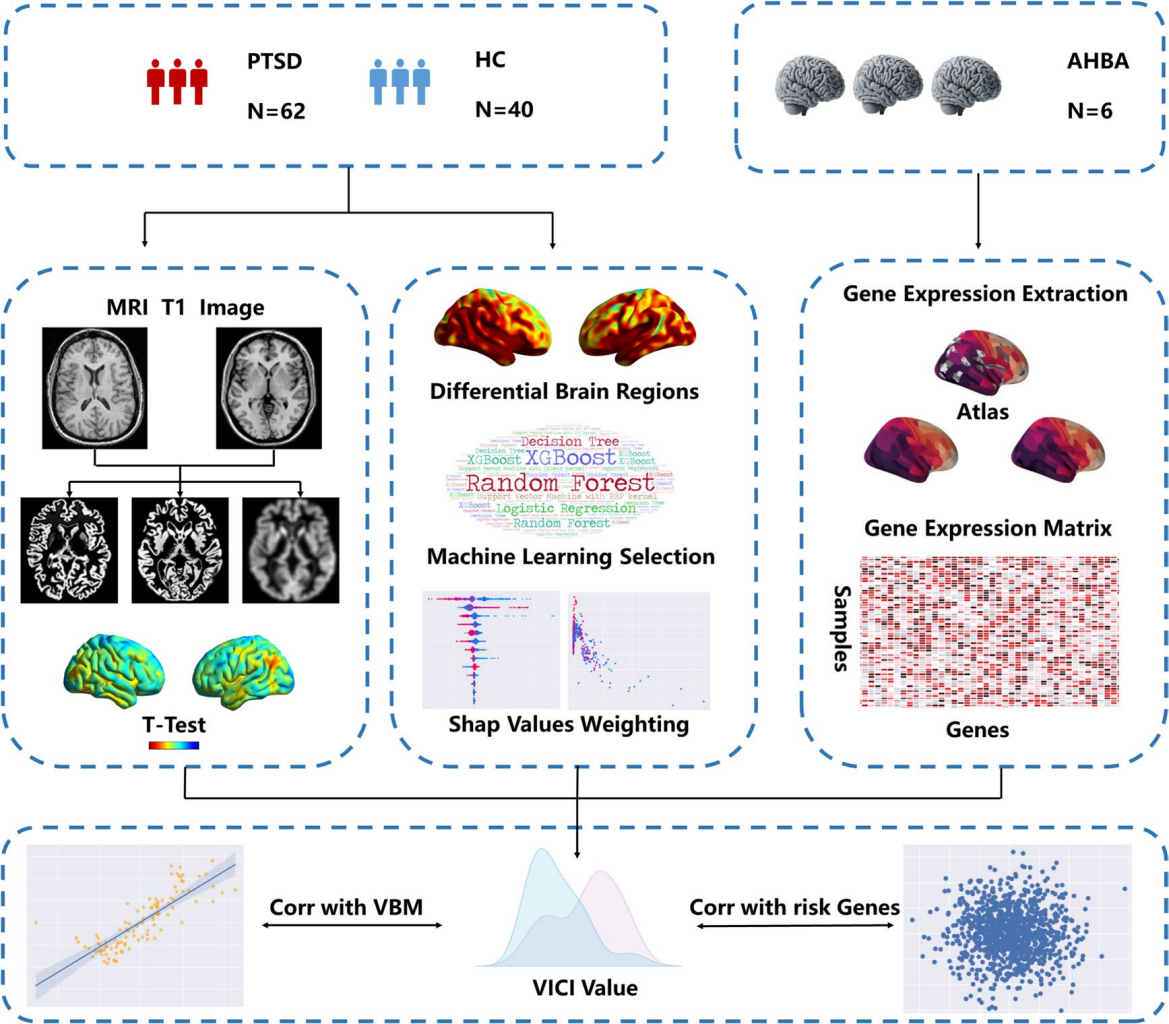
Flowchart of the study design. Abbreviations: VICI, Volumetric Integrated Classification Index; SHAP, SHapley Additive exPlanation; PTSD, post-traumatic stress disorder; VBM, voxel-based morphometry

An overview of the pipeline is shown in Fig. 1. The data used in this study were obtained from the Brain Aging in ADNI-DoD database and the ADNI database (adni.loni.usc.edu). A total of 102 individuals, aged between 60 and 80 years, were included in the study. Specifically, the PTSD data were sourced from the DOD-ADNI database, while the healthy control data were sourced from the ADNI database. Clinical assessment data including CAPSLIFE, MoCA, MMSE, and GDSCALE were downloaded for analysis. The PTSD group included 62 individuals who exhibited current and clinically significant symptoms of PTSD, as determined by CAPS-IV total severity scores. The healthy control group consisted of 40 individuals who had no history of exposure to traumatic events and did not present clinically significant symptoms related to PTSD.

### MRI Data Acquisition and Voxel-Based Morphometry

We utilized MRI scans conducted on a 3 T GE operating system. Participants underwent a standardized protocol involving high-resolution MRI T1 scans of the brain using a 3D IR-SPGR sequence. Images were downloaded from the ADNI-LONI database and checked for image quality. By using the MRICRON software [21], routine dcm2nii raw DICOM images of the participants were converted to Neuroimaging Informatics Technology Initiative (NIfTI) format for further processing. Next, the preprocessing steps were conducted using the Computational Anatomy Toolbox (CAT12; http://dbm.neuro.unijena.de/cat/) implemented in MATLAB 2016b (Math Works, Natick, MA, USA). 3D T1-weighted imaging scans were normalized with an affine, followed by nonlinear registration, corrected for bias field inhomogeneities. Then, all images were segmented into gray matter (GM), white matter (WM), and cerebrospinal fluid (CSF) components, using the tissue probability templates of European brain atlases [22]. After that, the segmented scans were normalized into the same template using the geodesic shooting algorithm. Multiplied by the nonlinear part of the deformation field, the normalized GM component was modulated to generate the relative gray matter volume (GMV). Then, a Gaussian kernel with an 8 mm full-width at half-maximum (FWHM) was applied to smooth the normalized GM images.

### VBM Calculation and Intergroup Comparison

Analysis for GMV values was conducted using SPM12 (Statistical Parametric Mapping; http://www.fil.ion.ucl.ac.uk/spm) based on MATLAB 2016b. Two sample t-tests were performed, with age and total intracranial volume (TIV) as covariates. Statistical significance was set at uncorrected voxel-wise *p* < 0.001 and family-wise error (FWE) corrected cluster-level *p* < 0.05.

To explore the correlations between the GMV values in brain regions with significant group differences and the total CAPS, Mini-Mental State Examination (MMSE), Montreal Cognitive Assessment (MoCA), and Geriatric Depression Scale (GDS) scores, Pearson correlation analyses were calculated using SPSS 24.0. The *p*-value < 0.05 was considered statistically significant.

### Classification Model Selection

In our quest to determine the optimal classification model, we utilized the scikit-learn library [23] to conduct a review and testing of previously employed MRI-based PTSD classification machine learning models. The selected candidate classifiers included Support Vector Machine (SVM) with linear and RBF kernels, Random Forest (RF), Logistic Regression (LR), Decision Tree, and Extreme Gradient Boosting (XGBoost). The dataset was split into a training set and a test set using a 9:1 ratio. To fine-tune the hyperparameters of each classifier, we performed embedded tenfold cross-validation on the training dataset. Additionally, to assess the statistical significance of the classification performance, we conducted 1000 permutation tests. This approach allowed us to evaluate the robustness of the classification results by comparing the true model performance against the performance obtained by random label permutations. Finally, we compared the classification performance of different classifiers on the test dataset.

Based on the VBM study, we conducted SHAP analysis [19] and applied it to the weighted sum of features to obtain a composite index [24, 25]. The SHAP method, based on cooperative game theory [26], measures the average marginal contribution of each feature to the prediction of the entire feature vector set. We utilized a weighted summing approach of SHAP values to comprehensively assess the involved features, resulting in a single value representing our volumetric integrated classification index (VICI) for the VBM data. Then, we compared the classification performance of the tested optimal machine learning model with the VICI predictions.

### Interpretability Evaluation

To evaluate the interpretability of the proposed framework, we tested the potential associations between the derived VICI values and three aspects of PTSD biological characterization:

### Associations Between VICI Changes and Brain Structural Abnormality Levels

We conducted two-sample t-tests to compare the disparities in brain morphological features between the PTSD and HC groups. The absolute *t*-values of these features were employed to signify the extent of structural brain abnormalities in PTSD. Additionally, we assessed the distinctions in VICI values between the two participant groups and computed absolute *t*-values for VICI to depict VICI alterations in PTSD, with age and TIV as covariates (uncorrected voxel-wise *p* < 0.001, FWE-corrected cluster-level *p* < 0.05).

### Associations Between VICI Values and Clinical Features in PTSD Patients

We conducted Pearson correlation analyses using SPSS 24.0 to investigate the relationships between the VICI values and total scores of the CAPS, MMSE, MoCA, and GDS. A *p*-value < 0.05 was considered statistically significant.

### Associations Between VICI Changes and Brain Expression Profiles of Risk Genes of PTSD

The Allen Human Brain Atlas dataset (AHBA, http://human.brain-map.org/) contains microarray expression data collected from six healthy donors (mean age = 42.50 ± 13.38 years, male/female = 5/1) with 3702 spatially distinct samples. AHBA samples were aligned to 246 parcels of the BNA brain atlas using the abagen toolbox (https://www.github.com/netneurolab/abagen). The preprocessing of gene expression data involved several steps [27]: (1) updating probe-to-gene annotations using information from Arnatkeviciute et al.; (2) filtering probes with intensities less than background in > 50% of samples; (3) selecting representative probes for each gene based on differential stability; (4) mapping tissue samples to regions defined in the BNA brain atlas; (5) normalizing sample expression values for each sample and donor across genes; (6) normalizing gene expression values for each gene and donor across all expression samples; (7) aggregating samples within regions based on matches from step 4; (8) filtering gene sets based on differential stability, selecting only the top 50% of genes with the highest differential stability score. PTSD risk genes were sourced from a PGC-GWAS study. After applying the pre-processing criteria, genes that met the inclusion criteria were included in this study. A two-sample t-test was utilized to calculate the *t*-value representing differences in VICI characteristics between the PTSD and HC groups at each brain site, with the mean *t*-value of brain mass obtained for each site. Genes potentially associated with changes in VICI were identified through Spearman’s correlation analysis between the mean *t*-value of VICI differences and mRNA expression levels of PTSD risk genes in brain regions. The *p*-value < 0.05 was considered statistically significant (Bonferroni correction).

## Results

### Demographic and Clinical Characteristics

The study included only male participants, and there was no statistically significant difference in age between PTSD and HC.

### Abnormal Brain Structural Changes in PTSD Patients

Compared with HC, PTSD patients showed significantly decreased GMV in the left middle frontal gyrus, left superior frontal gyrus, right middle frontal gyrus, and right superior frontal gyrus (cluster-level FWE correction with *p* < 0.05) (Table 1).

**Table 1:** Regions of grey matter reduction in PTSD versus healthy controls

Contrasts	Region	Coordinates (MNI)			Voxel	T values
		X	Y	Z		
PTSD < HC	Left superior frontal gyrus	-4	52	19	836	-4.6312
	Left middle frontal gyrus				810	
	Right superior frontal	34	44	28	1071	-4.9514

### Association Between Differential GMV and Clinical Measures

The difference in GMV among PTSD patients is not correlated with clinical scales.

### Model Performance and Construction of the VICI

**Fig. 2:**
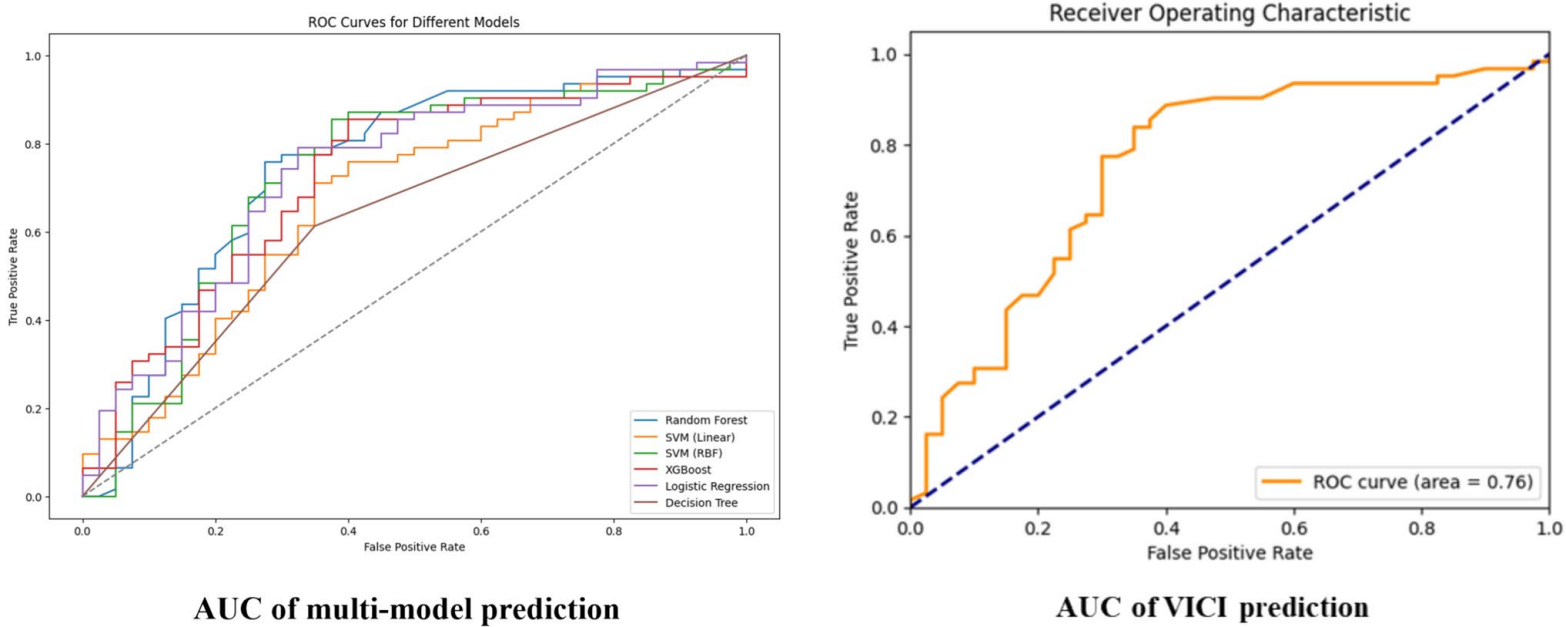
ROC curve of the multi-model and VICI prediction (***p*** < 0.05, averaged over 1000 permutation tests)

Using the 2498 differential brain regions obtained from VBM as input features, we assessed the performance of six machine learning algorithms: SVM with linear and RBF kernels, RF, LR, Decision Tree, and XGBoost. Our analysis revealed that RF emerged as the top performer, achieving an accuracy of 74%, specificity of 65%, sensitivity of 80%, and an ROC value of 0.765. SVM with RBF kernel closely followed RF, exhibiting an accuracy of 74%, specificity of 60%, sensitivity of 84%, and an ROC value of 0.719. XGBoost secured the third position with 73% accuracy, 60% specificity, 81% sensitivity, and an ROC value of 0.725. LR exhibited slightly lower performance, attaining an accuracy of 69%, specificity of 55%, sensitivity of 79%, and an ROC value of 0.735. SVM with linear kernel trailed LR, displaying a 65% accuracy, 42% specificity, 79% sensitivity, and an ROC value of 0.682. Lastly, Decision Tree performed the least effectively among the models, yielding a 63% accuracy, 60% specificity, 65% sensitivity, and an ROC value of 0.622 (Table 2). In summary, RF demonstrated superior performance across multiple metrics, making it the preferred choice for the subsequent analysis (Fig. 2).

**Table 2:** Comparison of classification performance among different machine learning models for PTSD diagnosis (*p* < 0.05, averaged over 1000 permutation tests)

Classifier	Accuracy	Specificity	Sensitivity	ROC Value	***P***-value
RF	74%	65%	80%	0.765	< 0.0001
SVM-RBF	74%	60%	84%	0.719	0.0010
XGBoost	73%	60%	81%	0.725	< 0.0001
LR	69%	55%	79%	0.735	< 0.0001
SVM-Linear	65%	42%	79%	0.682	0.0010
Decision Tree	63%	60%	65%	0.622	0.0030

By employing a weighted summing approach of SHAP values, we derived a VICI for the VBM data. This index provided a single value that comprehensively represented the predictive power of the included features. Subsequently, we compared the classification performance of the VICI with that of the optimized machine learning model. Notably, the classification efficacy of the VICI closely resembled that of the original RF model (see also Fig. 2).

### VICI Differences Between the PTSD and HC Groups

**Fig. 3:**
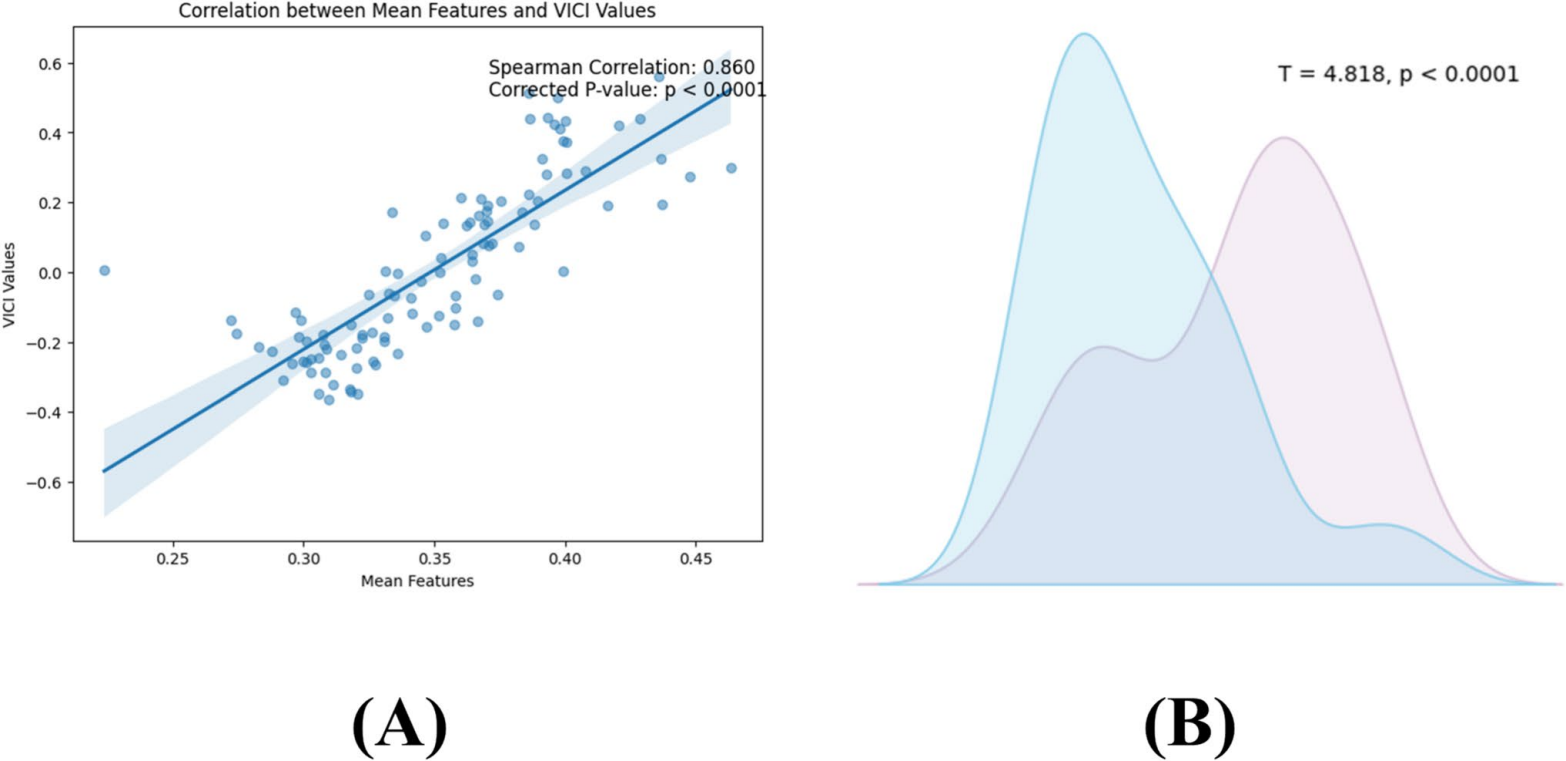
Group differences in VICI and its association with PTSD volumetric abnormalities. **A** The Spearman correlations in PTSD-related absolute changes between VICI and brain volumetric measures (*p* < 0.05). **B** The histogram distributions of VICI values in PTSD and HC and their differences

**Fig. 4:**
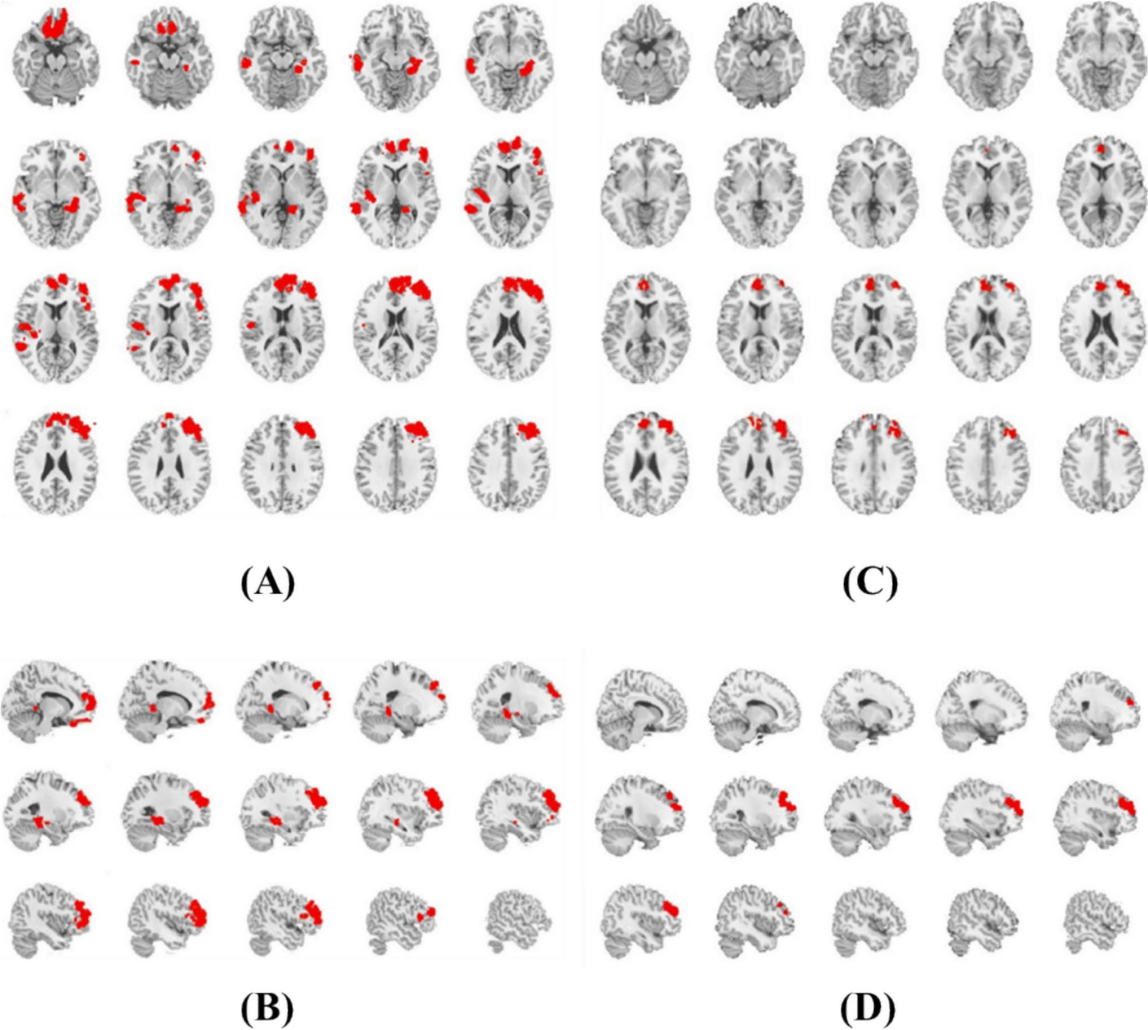
The intergroup difference in VICI values (**A**, **B**) and brain volumetric measures (**C**, **D**) between the PTSD and HC groups (*p* < 0.05, cluster FWE correction)

Firstly, we correlated the VICI values with the differential brain region values of the VBM and found a significant correlation (*r* = 0.86, *p* < 0.05) (Fig. 3). To explore the potential biological significance of VICI values, we compared the differences in VICI values between the PTSD group and the HC group using a two-sample t-test (Fig. 4). In comparison to the healthy control (HC) group, individuals with PTSD exhibited decreased VICI values for the following brain regions: right and left rectus, right parahippocampal and hippocampus, left medial temporal, right medial frontal, and left medial frontal gyrus (Table 3).

**Table 3:** Regions of VICI values reduction in PTSD versus healthy controls

Contrasts	Region	Coordinates (MNI)			Volume	T values
		X	Y	Z		
PTSD < HC	Right rectus	-4	31	-22	489	-4.6121
	Left Rectus				484	
	Right parahippocampal	24	-34	-7	302	-4.57
	Right hippocampus				268	
	Left medial Temporal	-57	-27	1	1270	-4.5165
	Right medial frontal gyrus	23	49	21	2833	-5.925
	Left medial frontal gyrus				1117	

### Association Between VICI and Clinical Measures

The VICI values among PTSD patients are not correlated with clinical scales.

### Association Between VICI and Brain Expression of Risk Genes of PTSD

Among the 34 PTSD-related risk genes [28], brain mRNA expression levels of 8 genes were found to be significantly associated with differences in VICI values between PTSD patients and HC by Spearman’s correlation analysis (*p* < 0.05, Bonferroni correction).

## Discussion

In this study, VBM was used to compare the GMV differences between patients with PTSD and HC. Notable reductions in GMV were observed in several prefrontal areas, including the left middle frontal gyrus, left superior frontal gyrus, medial sections, right middle frontal gyrus, and right superior frontal gyrus. These brain regions are associated with abnormalities in memory, emotion regulation, and fear processing [29], which are foundational to the core symptoms of PTSD. Moreover, our findings are consistent with previous studies [30–32], further reinforcing the theoretical link between these brain regions and PTSD.

The performance of six different machine learning algorithms was evaluated, with the RF model showcasing the best performance in classifying patients with PTSD, achieving the highest accuracy, specificity, sensitivity, and ROC value. Even though SVM with a RBF kernel and XGBoost have shown competitive performance, the stability and high accuracy rate of the RF model indicates that it is more reliable in handling structured large-scale neuroimaging datasets. This may be due to the RF model’s ability not to overfit when processing data with complex intrinsic structures and to better capture interactions among features [33].

Although black-box machine learning models have been proven superior to traditional clinical practices in many fields, their poor interpretability severely limits their clinical application [34, 35]. This presents a dilemma regarding the complexity and interpretability of ML models: on one hand, models are expected to be as simple as possible, potentially sacrificing performance; on the other hand, there is a desire for more powerful models, but increasing complexity may come at the cost of interpretability. To resolve the problem, we proposed a solitary scalar called volumetric integrated classification index—VICI—formulated by the weighted summation of SHAP values, to provide a single, comprehensive assessment of the predictive capability aggregated from the VBM data. Upon comparison with the optimized RF model, the VICI demonstrated classification efficacy that closely matched that of the RF model. This suggests that the VICI can serve as a simplified index to reflect the predictive power of complex machine learning models, yet having better interpretability and potential clinical application value.

The investigation into the VICI differences between the PTSD group and HC using a two-sample t-test revealed notable findings. Individuals with PTSD demonstrated decreased VICI values in several brain regions, specifically the right and left rectus, right parahippocampal and hippocampus, left medial temporal, right medial frontal, and left medial frontal gyrus, which is in line with earlier findings that persons with PTSD have structural anomalies in their brains [36–38]. These differences showed the VICI values could have significant implications in understanding the biological underpinnings of PTSD. Given that the regions mentioned mostly pertain to the limbic system and prefrontal areas that are strongly implicated in emotional processing and memory, the observed decrements could reflect the structural and functional abnormalities within neural circuits that underlie PTSD pathophysiology.

Further, our analysis delineates a molecular dimension to the PTSD phenotype by identifying a correlation between brain mRNA expression levels of 8 PTSD-related risk genes and variations in VICI values. The implicated genes are NRXN1, EXD3, CAMKV, IP6K1, TCF4, DCAF5, IMMP2L, and PGPEP1. The Spearman’s correlation analysis—corrected for multiple comparisons via the rigorous Bonferroni correction—suggests that these genes may play a role in the neurological changes observed in PTSD, possibly through mechanisms governing synaptic integrity (NRXN1), neuronal signaling (CAMKV, IP6K1) [39, 40], and transcriptional regulation (TCF4).

Particularly, NRXN1 (Neurexin 1) [41] is a presynaptic cell adhesion molecule involved in synaptic formation and function. Its association with VICI differences hints at synaptic alterations that might contribute to the neurobiological dysfunction in PTSD. Similarly, TCF4 (Transcription Factor 4) [42] plays a critical role in nervous system development, and dysregulation could have downstream effects resulting in the observed phenotypic differences in VICI values.

The integration of classification indices derived from neuroimaging with molecular genetics holds promise for elucidating comprehensive biomarkers for PTSD. The significant relationship between the VICI values and gene expression within critical brain regions underscores the potential of VICI as a biomarker reflecting the underlying genetic predisposition and the associated neuroanatomical manifestations of PTSD.

Expanding on the findings of this investigation, a positive discussion on implementation and future course might highlight the following paths. Expanding sample numbers and conducting multi-center studies are important ways to strengthen the generalizability of our findings about the relationship between specific brain regions’ changes in GMV, cerebral VICI scores, and PTSD. Furthermore, additional research into the genetic influence on brain structure is required in light of the connections found between the NRXN1 and TCF4 genes and changes in VICI values. This might help to explain how these genes contribute to the pathophysiology of PTSD.

Additionally, long-term research tracking these alterations may provide prognostic information about how the illness will develop and respond to therapy, guiding the development of individualized preventative and therapeutic plans. Practically speaking, the VICI has the potential to develop into an innovative diagnostic tool for PTSD that provides individualized diagnostic data. Furthermore, by examining VICI values and related gene expression, medical professionals may be better equipped to assess a patient’s prognosis and pathology, which will help them choose the best course of action.

These alterations could potentially be used as a gauge to assess how well different therapy approaches for treating PTSD work. This would then provide insight into early-stage, effective treatment approaches, allowing for necessary modifications to therapy protocols. In summary, VBM analysis and machine learning models have shown distinct neuroimaging and genetic features in PTSD patients.

### Limitation

This study, while providing some insight into the neurobiological basis of PTSD, has several limitations that must be addressed. First, the specificity of the machine learning models used for PTSD classification was not satisfactory across any of the models. While models such as Random Forest and SVM with RBF kernel achieved high sensitivity in identifying PTSD patients, their ability to accurately distinguish between PTSD patients and healthy controls was only partial. This lack of specificity could lead to false positives, limiting the practical clinical application of these models. Addressing this issue requires the exploration of additional features or the integration of multi-modal data, such as neurochemical or physiological biomarkers, to enhance the models’ performance and improve both sensitivity and specificity. Additionally, the study’s sample composition further limits the generalizability of the findings. The exclusive inclusion of male participants restricts the applicability of the results to other genders, and the relatively older age of participants, along with their specific trauma histories, means the results may not be representative of younger individuals or those exposed to different types of trauma. Future studies should aim to include a more diverse sample to explore potential variations in PTSD-related neurobiological mechanisms across gender, age, and trauma types. Moreover, the cross-sectional design of this study prevents an understanding of how PTSD-related brain changes evolve over time. Longitudinal studies are needed to track brain structure changes and their relationship with clinical outcomes, potentially providing insights into the progression of PTSD and the effectiveness of interventions. The Volumetric Integrated Classification Index (VICI), though promising as a potential diagnostic tool, also requires further validation across multiple centers and populations to establish its reliability and clinical relevance. Finally, while this study identified significant associations between VICI and specific PTSD-related risk genes, the underlying mechanisms remain unclear. A more in-depth functional analysis of these genetic findings is necessary to better understand their role in PTSD pathophysiology. Additionally, integrating VICI with other biomarkers could provide a more comprehensive diagnostic framework, further enhancing the robustness and precision of PTSD diagnostics.

## Conclusion

Through VBM analysis and the application of machine learning models, this study has revealed neuroimaging and genomic characteristics of individuals with PTSD, proposing the VICI value as a novel metric for assessing potential biomarkers of the condition. Future research should continue to explore the implications of VICI values and how they can inform PTSD diagnostics, treatment, and prognostic evaluation.

## Data Availability

The data that support the findings of this study are available from ADNI and ADNI-DOD.
